# In Vitro Synergistic Effect of Lefamulin with Doxycycline, Rifampin, and Quinupristin/Dalfopristin Against Enterococci

**DOI:** 10.3390/microorganisms12122515

**Published:** 2024-12-06

**Authors:** Yu-Hong Min, Yong-ung Kim, Min Chul Park

**Affiliations:** 1 College of K-Bio Health, Daegu Haany University, Gyeongsan 38610, Republic of Korea; yhmin@dhu.ac.kr; 2 Department of Pharmaceutical Engineering, Daegu Haany University, Gyeongsan 38610, Republic of Korea; ykim@dhu.ac.kr; 3 College of Pharmacy and Inje Institute of Pharmaceutical Sciences and Research, Inje University, Gimhae 50832, Republic of Korea

**Keywords:** synergy, enterococci, lefamulin, doxycycline, rifampin, quinupristin/dalfopristin

## Abstract

The widespread resistance of enterococci to many commonly used antimicrobial agents is a growing concern. Given that the current treatment options for enterococcal infections are limited, the discovery of new therapies, including combination therapies, is necessary. We evaluated double-drug combinations of lefamulin with doxycycline, rifampin, and quinupristin/dalfopristin for in vitro synergy against strains of *Enterococcus faecium* (*E. faecium*) and *Enterococcus faecalis* (*E. faecalis*) by using checkerboard and time-kill assays. In the checkerboard assay, the synergistic effect of lefamulin with doxycycline and rifampin was observed in 29 (85.3%) and 33 (97.1%) of the 34 different *E. faecium* strains tested, respectively. These combinations also showed synergistic effects against 17 (94.4%) of the 18 different vancomycin-resistant *E. faecium* strains. Among the 33 different *E. faecalis* strains, the combination of lefamulin with doxycycline, quinupristin/dalfopristin, and rifampin displayed synergistic effects in 31 (93.9%), 26 (78.8%), and 20 (60.6%) strains, respectively. No antagonism was observed in any of the combinations. The time-kill assay confirmed the synergistic effects of all these combinations. These synergistic combinations exhibited bacteriostatic activity. Although lefamulin is not currently used to treat enterococcal infections, we suggest that these combinations may serve as alternative drug regimens.

## 1. Introduction

Species of *Enterococcus* are common members of commensal communities in the gastrointestinal and genital tracts of humans; however, they are also opportunistic and nosocomial pathogens that can cause a variety of infections. These bacteria commonly cause urinary tract, intra-abdominal, pelvic, and soft tissue infections as well as life-threatening infections such as endocarditis and bacteremia [1]. However, enterococci have intrinsic and acquired resistance to almost all clinically used antimicrobial agents [2,3]. They are intrinsically resistant to cephalosporins, clindamycin, trimethoprim, trimethoprim–sulfamethoxazole, fusidic acid, and to clinically achievable concentrations of aminoglycosides [4]. Additionally, *Enterococcus faecalis* is intrinsically resistant to the streptogramin quinupristin/dalfopristin [4]. In addition, enterococci have rapidly acquired resistance to erythromycin, chloramphenicol, tetracycline, quinolones, and glycopeptides, as well as high-level resistance to aminoglycosides [2,3]. In particular, vancomycin-resistant *E. faecium* was classified as a ‘high priority’ for the development of new antibiotics by the World Health Organization in 2017 because of limited treatment options [5]. β-Lactams such as ampicillin and penicillin remain the treatment of choice for enterococcal infections, and resistance to these antibiotics is, fortunately, uncommon in *E. faecalis* [2]. Nevertheless, new therapeutic approaches are still needed owing to the generally limited treatment options for enterococcal infections.

Lefamulin was the first pleuromutilin antibiotic to be administered systemically in humans. Both intravenous and oral formulations were approved by the US Food and Drug Administration in 2019 for the treatment of community-acquired bacterial pneumonia. Lefamulin has a broad spectrum of activity against Gram-positive bacteria, including *E. faecium*, but lacks activity against *E. faecalis* [6].

Identifying antibiotic drug interactions is necessary to predict the outcomes of antimicrobial treatment. Synergistic drug interactions can enhance therapeutic efficacy and reduce side effects [7,8]. Combining antibiotics is also a rational approach to minimize the evolution of resistance [8,9,10]. In previous studies, researchers have demonstrated the synergistic potential of lefamulin with other antimicrobials. It has been shown that the combination of lefamulin and doxycycline displayed a potential synergistic effect against vancomycin-resistant and linezolid-resistant *E. faecium* [11]. Lefamulin in combination with doxycycline and aztreonam exerts a synergistic effect against *Staphylococcus aureus* and *Streptococcus pneumoniae*, respectively [12]. In addition, other pleuromutilin valnemulin or retapamulin in combination with vancomycin showed synergistic activity against vancomycin-resistant *E. faecium* [13]. The pleuromutilins valnemulin, tiamulin, and retapamulin demonstrated synergy with tetracycline against *S*. *aureus* both in vitro and in vivo [14]. Valnemulin exhibited a synergistic effect with colistin against multidrug-resistant Gram-negative pathogens [15]. Tiamulin exhibited synergistic activity with spectinomycin against *Streptococcus suis* [16]. It was also demonstrated that retapamulin produces synergistic activity in combination with either erythromycin or quinupristin against *E. faecalis*. Some pleuromutilin derivatives exhibited synergy when combined with doxycycline and tetracycline against *Acinetobacter baumannii* and *S. suis* [17,18].

Although lefamulin is not currently used to treat enterococcal infections, to find out new potential treatment option, this study evaluated in vitro activity of lefamulin in combination with other antibiotics (doxycycline, rifampin, and quinupristin/dalfopristin) against *E. faecium* and *E. faecalis*.

## 2. Materials and Methods

### 2.1. Bacterial Strains and Antibiotics

A total of 33 different clinical isolates of *E. faecium*, including 18 different isolates of vancomycin-resistant *E. faecium*, and a total of 32 different clinical isolates of *E. faecalis*, were collected from Severance Hospital and Dongsan Medical Center, Republic of Korea. *E. faecium* ATCC 19434 and *E. faecalis* ATCC 29212 were also tested. All the strains were routinely maintained in brain heart infusion broth or agar (BHIB, BHIA; MB Cell, Seoul, Republic of Korea). Lefamulin was purchased from MedChem Express (Monmouth Junction, NJ, USA). Doxycycline, rifampin, ampicillin, penicillin, vancomycin, linezolid, levofloxacin, erythromycin, gentamicin, and streptomycin were purchased from Sigma-Aldrich (St. Louis, MO, USA). Quinupristin/dalfopristin was obtained from Handok (Seoul, Republic of Korea).

### 2.2. Determination of Minimum Inhibitory Concentration (MIC)

The MIC values were determined by applying the broth microdilution method [4,19] using Mueller Hinton broth II (MHB II; Becton Dickinson, Sparks, MD, USA). Bacterial suspensions with a turbidity equivalent to that of a 0.5 McFarland standard were prepared by suspending log-phase growth from BHIA in MHB II. Suspensions were further diluted with MHB II to obtain a final inoculum of 5 × 10^5^ CFU/mL. In *E. faecium*, the final drug concentrations ranged from 0.031 to 64 µg/mL for lefamulin, doxycycline, and rifampin. In *E. faecalis*, the final drug concentrations ranged from 0.063 to 128 µg/mL for lefamulin, 0.031 to 64 µg/mL for doxycycline and quinupristin/dalfopristin, and 0.004 to 32 µg/mL for rifampin. Plates were incubated for 20 h in ambient air at 35 °C. *E. faecalis* ATCC 29212 was used as the quality control strain. The results were interpreted according to the Clinical and Laboratory Standards Institute (CLSI) guidelines [4].

### 2.3. Determination of Fractional Inhibitory Concentration Index (FICI)

To elucidate the possible interaction between lefamulin and other antibiotics, checkerboard assays were performed as previously described [20]. In specific, the strains were grown in BHIA for 18 to 24 h. Direct suspensions of log-phase colonies were made in MHB II to 0.5 McFarland standard then diluted in MHB II to achieve a concentration of 1 × 10^6^ CFU/mL. Lefamulin was serially diluted two-fold along the rows of a 96-well polystyrene microplate, and the other drug was serially diluted two-fold along the columns. Next, 50 µL of the bacterial suspension containing 1 × 10^6^ CFU/mL was added to wells containing 50 µL of the antibiotic dilutions. Thus, the final inoculum size in each well was 5 × 10^5^ CFU/mL. The concentrations tested for each drug were chosen based on predetermined MICs. The final concentrations of each drug used in the combinations ranged from 1/32 × MIC to 2 × MIC. The positive control wells contained the bacterial suspension without antibiotics, while the negative control wells contained only media. Values of MICs of the drugs alone and in combination were determined as the lowest concentrations of the antibiotics that inhibit visible growth after overnight incubation for 24 h in ambient air at 35 °C. Each checkerboard assay was performed in duplicate. The interaction of the drugs in a combination was evaluated by the fractional inhibitory concentration (FIC) index (FICI). The FICI was calculated using the following equation:

$$FICI={FIC}_{A}+{FIC}_{B}=\frac{MIC\;of\;drug\;A\;in\;combination}{MIC\;of\;drug\;A\;alone}+\frac{MIC\;of\;drug\;B\;in\;combination}{MIC\;of\;drug\;B\;alone}$$

FICI results for each combination were interpreted as follows: synergy (FICI ≤ 0.5); no interaction (0.5 < FICI ≤ 4); antagonism (FICI > 4) [21].

### 2.4. Time-Kill Assay

To further study the activity of lefamulin in combination with other antibiotics, in vitro time-kill assays were performed. Two ATCC stains (*E. faecium* ATCC 19434 and *E. faecalis* ATCC 29212) and four clinical strains (*E. faecium* VRE19, *E. faecium* VRE42, *E. faecalis* 24, and *E. faecalis* 225) were tested against lefamulin (1/4 × MIC) and other antibiotics (1/4 × MIC) alone and in combination. The strains were grown in BHIA for 18 to 24 h. Freshly prepared colonies were resuspended in BHIB and were adjusted to produce a 0.5 McFarland standard. Bacterial suspensions were diluted in BHIB so that the final culture density in the tubes was equal to 5 × 10^5^ CFU/mL. The assay was carried out in 2 mL broth samples inoculated in the tubes. Positive control included the bacterial suspension without antibiotics and the negative control included only media without antibiotics. The cultures were aerobically incubated at 35 °C with continuous shaking at 150 rpm. Colony enumeration was performed at 0- and 24 h time points by removing 100 µL of the culture, diluting as appropriate, and plating 100 µL on BHIA plates. Colony count data (log_10_ CFU/mL) were expressed as the mean value of three independent replicates ± the standard deviation (SD). Statistical analysis of the significance differences between the mean values of the results were identified by one-way analysis of variance (ANOVA) followed by Tukey’s post hoc test at the 5% level of significance (*p* < 0.05) using the IBM SPSS Statistics, version 29.0 (IBM Corp., Armonk, NY, USA).

Synergy was defined as a ≥2 log_10_ CFU/mL decrease at 24 h for the combination when compared with the most active single agent [22]. Indifference was defined as a <2 log_10_ CFU/mL decrease or increase at 24 h for the combination when compared with the most active single agent, and antagonism was defined as a ≥2 log_10_ CFU/mL increase at 24 h for the combination when compared with the most active single agent [22]. Bactericidal activity was defined as a ≥3 log_10_ CFU/mL decrease at 24 h for the combination when compared with the initial inoculum [23].

## 3. Results

### 3.1. Susceptibility

Susceptibility of all the clinical strains (33 *E. faecium* and 32 *E. faecalis*) to clinically available antibiotics was analyzed by the broth microdilution. Resistance profiles are listed in Supplementary Tables S1 and S2. All *E. faecium* clinical strains were sensitive to linezolid. However, all but one of the *E. faecium* clinical strains were resistant to ampicillin, penicillin, levofloxacin, and erythromycin. A high incidence of resistance was detected for vancomycin (54.5%) and quinupristin/dalfopristin (51.5%) in *E. faecium* strains. High-level resistance to gentamicin was more frequently detected (93.9%) than high-level resistance to streptomycin (27.3%). In *E. faecalis*, more than 90% were susceptible to ampicillin, penicillin, and vancomycin. The majority of *E. faecalis* strains were also susceptible to linezolid. Resistance rates to levofloxacin and erythromycin were 46.9% and 40.6%, respectively. Rates of high-level resistance to gentamicin and streptomycin were 68.8% and 31.3%, respectively. Overall, 97.0% of *E. faecium* and 62.5% of *E. faecalis* were multidrug resistant (Tables S1 and S2, Table 1 and Table 2).

The MICs of the antibiotics used to assess synergy against *E. faecium* and *E. faecalis* clinical isolates are shown in Table 1 and Table 2, respectively. MICs of lefamulin against *E. faecium* clinical strains ranged from 0.063 to 32 μg/mL (Table 1). All but two of the *E. faecium* clinical strains (the exceptions being strains 95 and VRE99) were susceptible to doxycycline. Of the 33 different *E. faecium* clinical strains, 23 strains were resistant to rifampin. In contrast to the activity of lefamulin against *E. faecium*, the activity of lefamulin against *E. faecalis* was low, as indicated by the relatively high MIC values ranging from 32 to 128 μg/mL (Table 2). Of the 32 different *E. faecalis* clinical strains, 20 strains were resistant to doxycycline and 24 strains were resistant to rifampin. MICs of quinupristin/dalfopristin ranged from 4 to 64 μg/mL.

### 3.2. FICI Determination

Checkerboard assays were performed to determine the potential synergistic effects of combinations of lefamulin and other antibiotics. The FICI values of the combinations against *E. faecium* and *E. faecalis* are presented in Table 1 and Table 2, respectively. Synergistic effects were observed for lefamulin plus doxycycline and lefamulin plus rifampin in 29 (85.3%) and 33 (97.1%) of 34 tested *E. faecium* strains, respectively (Table 1). These combinations also showed synergistic effects against vancomycin-resistant *E. faecium* (94.4%). There was no antagonistic interaction in these combinations. In one of two doxycycline-resistant *E. faecium* strains tested, the addition of lefamulin reduced the MIC of doxycycline to the susceptible range (MIC ≤ 4 μg/mL). In all rifampin-resistant *E. faecium* strains, the addition of lefamulin resulted in a reduction in the MIC values of rifampin to the susceptible range (MIC ≤ 1 μg/mL). However, the combination of lefamulin and quinupristin/dalfopristin resulted in no interaction against *E. faecium* with FICI ranging from 0.625 to 2 μg/mL.

In *E. faecalis*, the combination of lefamulin with doxycycline, quinupristin/dalfopristin and rifampin showed synergistic effects in 31 (93.9%), 26 (78.8%), and 20 (60.6%) of 33 strains, respectively (Table 2). None of the combinations resulted in antagonism. The combination of lefamulin and doxycycline produced a significant rate of synergy against doxycycline-resistant strains (90%). The combination of lefamulin and rifampin also showed a high rate of synergy against rifampin-resistant strains (75%).

### 3.3. Time-Kill Assay

The specific log_10_CFU/mL changes in the combination of lefamulin with other antimicrobials at concentrations of 1/4 × MIC that were effective in the checkerboard assay against *E. faecium* and *E. faecalis* strains are shown in Table 3. In the case of *E. faecium*, the combination of lefamulin plus doxycycline and lefamulin plus rifampin showed synergistic effects, with a drop of ≥2 log_10_CFU/mL compared with the most active single agent. Meanwhile, the results indicate that the synergistic effects of these combinations were bacteriostatic. As for *E. faecalis*, a drop of ≥2 log_10_CFU/mL was also observed with the combination of lefamulin with doxycycline, rifampin, or quinupristin/dalfopristin, which indicates that these combinations are synergistic. All combinations showed bacteriostatic effects.

## 4. Discussion

In this study, we determined that lefamulin exhibited good activity against a number of *E. faecium* clinical strains tested (MIC_50_ = 0.125 μg/mL), which is consistent with previously reported findings [24,25]. The synergistic effects of lefamulin in combination with rifampin and doxycycline against *E. faecium*, including vancomycin-resistant *E. faecium*, were evident in vitro through checkerboard and time-kill assays. Synergism between lefamulin and doxycycline against vancomycin-resistant *E. faecium* had been shown in a previous study, in which the synergy prevalence was 33.3% [11]. However, a much higher synergy rate (94.4%) was found here in the vancomycin-resistant *E. faecium* isolates. Therefore, additional tests against strains with diverse epidemiological backgrounds are required to generalize the benefits of this combination. It is noteworthy to highlight that the combination of lefamulin and doxycycline restored doxycycline susceptibility in one (50%) of two doxycycline-resistant strains [one (100%) of the one strain that was resistant to both doxycycline and vancomycin], and lefamulin and rifampin restored rifampin susceptibility in 24 (100%) of 24 rifampin-resistant strains [as well as 11 (100%) of the 11 strains that were resistant to both rifampin and vancomycin].

Lefamulin exhibited low activity against *E. faecalis* in contrast to activity against *E. faecium* (MIC_50_ = 64 μg/mL), which is consistent with previous reports [24,25]. However, synergistic effects were observed in combinations of lefamulin with quinupristin/dalfopristin, as well as doxycycline and rifampin, as evidenced by the results of the checkerboard and time-kill assays. Synergism with quinupristin/dalfopristin was also observed for another pleuromutilin retapamulin [20]. Thus, it seems likely that the synergism between lefamulin and quinupristin/dalfopristin was due to the synergism between lefamulin and quinupristin and not dalfopristin, as was the case with retapamulin [20]. Synergism between retapamulin and erythromycin has also been previously reported [20]; however, the combination of lefamulin and erythromycin was not tested in the present study because it is contraindicated [26]. Coadministration of lefamulin and erythromycin can prolong QT interval, which confers an increased risk of arrhythmia [27]. The combination of lefamulin with doxycycline and rifampin increased drug susceptibility, demonstrating synergistic effects in 90% and 75% of the doxycycline-resistant and rifampin-resistant strains, respectively.

The results of this microbiological study suggest that the combination of lefamulin and rifampin displayed good synergism against both *E. faecalis* and *E. faecium*, including vancomycin-resistant *E. faecium*. However, clinical use of this combination may be challenging because concomitant administration of lefamulin with rifampin can decrease plasma concentration of lefamulin [26]. Therefore, the synergistic effects of lefamulin and rifampin observed in this study require clinical validation.

Like other pleuromutilins, lefamulin inhibits bacterial protein synthesis by binding to the A- and *p*-sites of the peptidyl transferase center (PTC) of the 50S ribosomal subunit [28]. The tetracycline group of antibiotics such as doxycycline also inhibits protein synthesis by binding to the 16S rRNA of the 30S ribosomal subunit [29]. Thus, we believe that the mechanism of synergy between lefamulin and doxycycline conforms to the “parallel pathway inhibition model”, which suggests that the two drugs are synergistic if they inhibit two targets on parallel pathways essential for an observed phenotype, in this case, protein synthesis at the ribosome [30,31]. Quinupristin binds to the exit tunnel adjacent to the PTC [32]. Therefore, direct physical interactions between lefamulin and quinupristin may occur at the target and induce mutual stabilization of their binding, as is the case in synergism between quinupristin and dalfopristin [31,33]. Rifampin inhibits RNA polymerase and causes a decrease in the level of 23S rRNA, which is a target of lefamulin [34]. Lefamulin may also decrease the translation of RNA polymerase, the target of rifampin. Taken together, these two mechanisms could potentially lower the concentration of each drug required to exert antimicrobial activity. Further studies are required to test these hypotheses.

The present study demonstrates the synergistic effects of lefamulin and several groups of antibiotics. As demonstrated in previous studies, other pleuromutilins have synergistic potential with other groups of antibiotics such as glycopeptides and aminoglycosides [13,16]. Therefore, further investigations are required to determine the potential synergism of lefamulin with other antibiotics, particularly ampicillin and penicillin, which are the preferred drugs for the treatment of enterococcal infections.

## 5. Conclusions

The results of in vitro assays reported here indicate that combinations of lefamulin with other antibiotics (specifically lefamulin and doxycycline or lefamulin and rifampin) display synergistic effects against *E. faecium*, including vancomycin-resistant *E. faecium*. Additionally, the combinations of lefamulin with doxycycline, rifampin, or quinupristin/dalfopristin displayed potential synergistic effects against *E. faecalis*. Although lefamulin is not currently used to treat enterococcal infections, treatment regimens based on these combinations may provide an alternative therapeutic strategy. However, it is noteworthy to emphasize that the synergistic effects of these combinations were bacteriostatic. With prolonged exposure, there is a potential for growth to be no longer inhibited. Therefore, these results should be interpreted with caution. Also, these in vitro results need to be confirmed in animal and clinical studies.

## Figures and Tables

**Table 1:** Synergistic activities of lefamulin with doxycycline or rifampin against *Enterococcus faecium* strains.

Strains ^a^	MIC (μg/mL) Alone			LEF + DOX		LEF + RIF	
	LEF	DOX	RIF	MIC in Combination	FICI ^b^	MIC in Combination	FICI ^b^
ATCC	0.063	0.25	16	0.016/0.063	**0.5**	0.016/1	**0.313**
3	0.25	0.25	8	0.063/0.063	**0.5**	0.031/0.5	**0.188**
4	0.125	8	8	0.031/1	**0.375**	0.016/0.5	**0.188**
5	0.063	0.25	2	0.031/0.063	0.75	0.008/0.25	**0.25**
20	0.125	0.25	16	0.016/0.063	**0.375**	0.016/1	**0.188**
26	0.063	8	4	0.016/2	**0.5**	0.016/0.25	**0.313**
36	0.063	0.063	0.5	0.031/0.008	0.625	0.016/0.063	**0.375**
37	32	0.25	0.063	1/0.063	**0.281**	1/0.008	**0.156**
44	0.125	0.125	8	0.031/0.031	**0.5**	0.016/1	**0.25**
57	64	0.25	8	4/0.063	**0.313**	0.25/1	**0.129**
58	0.125	0.125	8	0.031/0.016	**0.375**	0.016/1	**0.25**
59	0.125	0.25	8	0.031/0.031	**0.375**	0.016/1	**0.25**
60	0.125	0.125	16	0.063/0.016	0.625	0.016/0.5	**0.156**
61	0.125	0.125	8	0.016/0.031	**0.375**	0.016/0.5	**0.188**
93	0.125	0.25	8	0.031/0.031	**0.375**	0.016/1	**0.25**
95	64	32	4	8/16	0.625	0.5/1	**0.258**
VRE31	0.125	0.125	8	0.031/0.031	**0.5**	0.016/0.5	**0.188**
VRE36	0.063	2	2	0.016/0.25	**0.375**	0.016/0.125	**0.313**
VRE40	0.063	2	8	0.016/0.25	**0.375**	0.008/1	**0.25**
VRE19	0.125	0.25	8	0.031/0.031	**0.375**	0.016/1	**0.25**
VRE34	0.063	0.125	4	0.016/0.031	**0.5**	0.008/0.5	**0.25**
VRE35	0.125	0.063	1	0.031/0.016	**0.5**	0.031/0.063	**0.313**
VRE41	0.125	0.25	1	0.063/0.031	0.625	0.031/0.063	**0.313**
VRE42	0.063	0.25	2	0.016/0.031	**0.375**	0.016/0.125	**0.313**
VRE43	0.063	0.063	1	0.016/0.016	**0.5**	0.016/0.063	**0.313**
VRE46	0.125	0.125	4	0.031/0.031	**0.5**	0.016/0.5	**0.25**
VRE48	0.063	0.063	1	0.016/0.016	**0.5**	0.008/0.125	**0.25**
VRE75	0.063	0.063	8	0.016/0.016	**0.5**	0.016/0.5	**0.313**
VRE80	0.063	0.125	2	0.016/0.031	**0.5**	0.008/0.25	**0.25**
VRE99	0.125	16	8	0.016/2	**0.25**	0.008/0.5	**0.125**
VRE84	0.25	0.125	16	0.063/0.016	**0.375**	0.031/1	**0.188**
VRE96	0.125	0.125	16	0.031/0.016	**0.375**	0.063/0.25	0.516
VRE97	0.125	0.25	16	0.016/0.063	**0.375**	0.031/1	**0.313**
VRE98	0.125	0.25	16	0.031/0.063	**0.5**	0.008/1	**0.125**

LEF, lefamulin; DOX, doxycycline; RIF, rifampin. ^a^ ATCC, *E. faecium* ATCC 19434; VRE, vancomycin-resistant *E. faecium*. ^b^ Synergistic results (FICI ≤ 0.5) are indicated in bold.

**Table 2:** Synergistic activities of lefamulin with doxycycline, rifampin, or quinupristin/dalfopristin against *E. faecalis* strains.

Strains ^a^	MIC (μg/mL) Alone				LEF + DOX		LEF + RIF		LEF + Q/D	
	LEF	DOX	RIF	Q/D	MIC in Combination	FICI ^b^	MIC in Combination	FICI ^b^	MIC in Combination	FICI ^b^
ATCC	32	4	1	4	1/1	**0.281**	4/0.25	**0.375**	4/1	**0.375**
951	128	8	4	16	8/2	**0.313**	32/2	0.75	8/4	**0.313**
573	32	16	4	8	2/4	**0.313**	1/1	**0.281**	4/2	**0.375**
23	64	0.5	0.031	8	1/0.125	**0.266**	1/0.016	0.516	4/2	**0.313**
24	128	0.5	16	8	1/0.125	**0.258**	1/4	**0.258**	4/2	**0.281**
114	128	0.5	4	4	1/0.125	**0.258**	2/1	**0.266**	4/1	**0.281**
154	32	16	2	4	2/4	**0.313**	2/1	0.563	4/1	**0.375**
940	64	16	16	4	1/4	**0.266**	1/8	0.516	8/0.5	**0.25**
196	64	16	4	4	1/4	**0.266**	1/0.5	**0.141**	2/1	**0.281**
507	64	0.5	4	4	2/0.125	**0.281**	1/2	0.516	8/1	**0.375**
846	64	16	2	4	2/4	**0.281**	8/0.5	**0.375**	4/1	**0.313**
509	32	16	8	4	1/4	**0.281**	1/2	**0.281**	8/0.5	**0.375**
365	64	32	2	4	8/8	**0.375**	4/1	0.563	16/0.25	**0.313**
293	128	32	2	8	1/16	0.508	1/1	0.508	4/2	**0.281**
536	64	0.5	16	8	1/0.125	**0.266**	1/4	**0.266**	4/2	**0.313**
709	64	32	16	8	1/8	**0.266**	1/4	**0.266**	4/2	**0.313**
269	64	16	4	4	16/4	**0.5**	4/1	**0.313**	8/1	**0.375**
232	64	0.5	4	8	2/0.125	**0.281**	16/1	**0.5**	4/2	**0.313**
60	64	0.5	32	4	1/0.125	**0.266**	2/4	**0.156**	8/1	**0.375**
508	64	32	4	4	2/4	**0.156**	4/1	**0.313**	4/1	**0.313**
103	128	32	8	16	16/8	**0.375**	4/2	**0.281**	32/4	**0.5**
225	64	16	4	16	1/4	**0.266**	1/1	**0.266**	4/2	**0.188**
861	128	16	4	16	32/8	0.75	4/2	0.531	32/2	**0.375**
105	128	8	2	32	32/2	**0.5**	64/1	1	32/16	0.75
720	128	32	4	16	4/8	**0.281**	16/2	0.625	64/8	1
7	64	8	1	8	4/2	**0.313**	2/0.5	0.531	4/2	**0.313**
466	64	16	8	8	1/4	**0.266**	32/4	1	4/1	**0.188**
907	128	32	8	16	2/8	**0.266**	32/2	**0.5**	32/8	0.75
800	128	16	4	16	32/4	**0.5**	32/1	**0.5**	16/8	0.625
188	128	16	16	16	32/4	**0.5**	2/4	**0.266**	32/8	0.75
110	128	16	16	16	8/4	**0.313**	1/4	**0.258**	32/8	0.75
665	64	0.25	0.008	8	8/0.06	**0.375**	8/0.004	0.625	8/1	**0.25**
564	128	8	4	64	2/2	**0.266**	4/1	**0.281**	4/32	0.531

LEF, lefamulin; DOX, doxycycline; RIF, rifampin; Q/D, quinupristin/dalfopristin. ^a^ ATCC, *E. faecalis* ATCC 29212. ^b^ Synergistic results (FICI ≤ 0.5) are indicated in bold.

**Table 3:** The log_10_CFU/mL and log changes between the combination and the most active antibiotic after incubation for 24 h.

Strains	Antibiotics	log_10_CFU/mL	Changes vs. Most Active Antibiotic
*E. faecium* ATCC 19434	-	8.50 ± 0.20 ^ab^	
	LEF	8.49 ± 0.11 ^ab^	
	DOX	8.62 ± 0.19 ^a^	
	RIF	8.13 ± 0.16 ^b^	
	LEF + DOX	6.15 ± 0.18 ^c^	−2.33
	LEF + RIF	5.74 ± 0.24 ^c^	−2.39
*E. faecium* VRE19	-	9.04 ± 0.03 ^a^	
	LEF	8.42 ± 0.01 ^ab^	
	DOX	8.31 ± 0.32 ^ab^	
	RIF	8.04 ± 0.06 ^b^	
	LEF + DOX	5.58 ± 0.26 ^c^	−2.72
	LEF + RIF	5.17 ± 0.24 ^c^	−2.87
*E. faecium* VRE42	-	8.84 ± 0.03 ^a^	
	LEF	8.52 ± 0.01 ^a^	
	DOX	8.37 ± 0.20 ^a^	
	RIF	8.35 ± 0.04 ^a^	
	LEF + DOX	5.79 ± 0.02 ^b^	−2.59
	LEF + RIF	5.49 ± 0.26 ^b^	−2.86
*E. faecalis* ATCC 29212	-	8.70 ± 0.22 ^a^	
	LEF	9.02 ± 0.02 ^a^	
	DOX	8.61 ± 0.22 ^a^	
	RIF	8.57 ± 0.30 ^a^	
	Q/D	8.79 ± 0.27 ^a^	
	LEF + DOX	5.48 ± 0.38 ^b^	−3.12
	LEF + RIF	6.01 ± 0.27 ^b^	−2.56
	LEF + Q/D	5.57 ± 0.38 ^b^	−3.22
*E. faecalis* 24	-	8.89 ± 0.22 ^a^	
	LEF	8.87 ± 0.07 ^a^	
	DOX	9.06 ± 0.02 ^a^	
	RIF	7.94 ± 0.14 ^b^	
	Q/D	8.81 ± 0.24 ^a^	
	LEF + DOX	5.30 ± 0.12 ^c^	−3.57
	LEF + RIF	5.81 ± 0.10 ^c^	−2.13
	LEF + Q/D	5.59 ± 0.36 ^c^	−3.21
*E. faecalis* 225	-	8.94 ± 0.16 ^a^	
	LEF	8.97 ± 0.17 ^a^	
	DOX	9.30 ± 0.06 ^a^	
	RIF	8.11 ± 0.33 ^b^	
	Q/D	8.84 ± 0.31 ^a^	
	LEF + DOX	5.02 ± 0.06 ^d^	−3.94
	LEF + RIF	5.89 ± 0.12 ^c^	−2.22
	LEF + Q/D	5.04 ± 0.12 ^d^	−3.80

LEF, lefamulin; DOX, doxycycline; RIF, rifampin; Q/D, quinupristin/dalfopristin. 1/4 × MICs of all antibiotics were used in the time-kill assays. Values in each strain with different superscript letters are significantly different at *p* < 0.05.

## Data Availability

The original contributions presented in the study are included in the article/Supplementary Materials, further inquiries can be directed to the corresponding author.

## Supplementary Materials

The following supporting information can be downloaded at: https://www.mdpi.com/article/10.3390/microorganisms12122515/s1, Table S1: Antimicrobial resistance pattern of *E. faecium* strains; Table S2: Antimicrobial resistance pattern *E. faecalis* strains.
