# Supplementary material for: In Vitro Synergistic Effect of Lefamulin with Doxycycline, Rifampin, and Quinupristin/Dalfopristin Against Enterococci
Source: Microorganisms. 2024 Dec 6;12(12):2515. doi: 10.3390/microorganisms12122515 (PMC11678898; doi:10.3390/microorganisms12122515)
Supplement: Supplementary file 1 [file microorganisms-12-02515-s001.zip › microorganisms-3325327-supplementary.pdf]

Table S1. Antimicrobial resistance pattern of *E. faecium* strains.

Strains	AMP	PEN	VAN	LZD	Q/D	LEV	ERY	HLGR	HLSR
3	R	R	S	S	R	R	R	+	+
4	R	R	S	S	R	R	R	+	-
5	R	R	S	S	R	R	R	+	-
20	R	R	S	S	R	R	R	+	-
26	R	R	S	S	R	R	R	+	+
36	R	R	S	S	R	R	R	+	-
37	S	S	S	S	R	S	I	-	-
44	R	R	S	S	R	R	R	+	-
57	R	R	S	S	R	R	R	+	+
58	R	R	S	S	I	R	R	+	-
59	R	R	S	S	I	R	R	+	-
60	R	R	S	S	I	R	R	+	+
61	R	R	S	S	I	R	R	+	-
93	R	R	S	S	I	R	R	+	-
95	R	R	S	S	R	R	R	+	+
VRE31	R	R	R	S	R	R	R	+	+
VRE36	R	R	R	S	R	R	R	+	-
VRE40	R	R	R	S	I	R	R	+	-
VRE19	R	R	R	S	R	R	R	+	-
VRE34	R	R	R	S	I	R	R	+	-
VRE35	R	R	R	S	R	R	R	+	-
VRE41	R	R	R	S	R	R	R	+	+
VRE42	R	R	R	S	R	R	R	+	+
VRE43	R	R	R	S	I	R	R	+	-
VRE46	R	R	R	S	R	R	R	+	-
VRE48	R	R	R	S	I	R	R	+	-
VRE75	R	R	R	S	I	R	R	+	-
VRE80	R	R	R	S	I	R	R	+	-
VRE99	R	R	R	S	I	R	R	-	-
VRE84	R	R	R	S	I	R	R	+	-
VRE96	R	R	R	S	S	R	R	+	-
VRE97	R	R	R	S	I	R	R	+	-
VRE98	R	R	R	S	I	R	R	+	+

AMP, ampicillin; PEN, penicillin; VAN, vancomycin; LZD, linezolid; Q/D, quinupristin/dalfopristin; LEV, levofloxacin; ERY, erythromycin; HLGR, high-level gentamicin resistance; HLSR, high-level streptomycin resistance; R, resistant; I, intermediate; S, susceptible.

Table S2. Antimicrobial resistance pattern of *E. faecalis* strains.

Strains	AMP	PEN	VAN	LZD	LEV	ERY	HLGR	HLSR
951	S	S	S	S	R	S	+	-
573	S	S	S	S	S	S	-	-
23	S	S	S	S	R	S	+	-
24	S	S	S	S	R	S	+	-
114	S	S	S	S	S	S	-	-
154	S	S	S	S	S	I	-	-
940	S	S	S	S	R	I	-	-
196	S	S	S	S	R	I	-	-
507	S	S	S	I	R	I	+	-
846	S	S	S	I	S	I	+	-
509	S	S	S	S	S	I	+	-
365	S	S	S	S	I	I	-	-
293	S	S	S	S	S	I	+	+
536	S	S	S	I	S	I	+	-
709	S	S	S	S	S	I	-	-
269	S	S	S	S	S	I	-	-
232	S	S	S	S	S	I	+	-
60	S	S	S	S	S	I	-	-
508	S	S	S	S	R	I	-	-
103	S	S	S	S	R	R	+	+
225	S	S	S	S	I	R	+	-
861	S	S	S	S	R	R	+	+
105	S	R	S	S	R	R	+	-
720	S	S	S	S	R	R	+	+
7	S	S	S	S	R	R	+	+
466	S	S	S	S	R	R	+	-
907	S	S	S	S	I	R	+	+
800	S	S	S	S	S	R	+	+
188	S	S	S	S	S	R	+	+
110	S	S	S	S	I	R	+	+
665	S	S	S	I	R	R	+	+
564	R	R	R	S	R	R	+	-

AMP, ampicillin; PEN, penicillin; VAN, vancomycin; LZD, linezolid; LEV, levofloxacin; ERY, erythromycin; HLGR, high-level gentamicin resistance; HLSR, high-level streptomycin resistance; R, resistant; I, intermediate; S, susceptible.
